# Fluorescence-guided laparoscopic lymph node biopsy for lymphoma: the FLABILY study

**DOI:** 10.1007/s13304-024-01909-0

**Published:** 2024-06-14

**Authors:** Marco Casaccia, Giovanni Alemanno, Paolo Prosperi, Graziano Ceccarelli, Stefano Olmi, Alberto Oldani, Mauro Santarelli, Roberta Tutino, Franco De Cian

**Affiliations:** 1 Department of Surgical Sciences and Integrated Diagnostics (DISC), University of Genoa, Genoa, Italy; 2 Surgical Clinic I Unit, IRCCS San Martino Hospital, IST Monoblocco XI Piano - Largo Rosanna Benzi, 10 16132, Genoa, Italy; 3 Unit of Emergency Surgery, Careggi University Hospital, Florence, Italy; 4 General Surgery Department, ASL 2 Umbria, San Giovanni Battista Hospital, Foligno, Italy; 5 Università Vita-Salute San Raffaele, Milan, Italy; 6 Department of General and Oncological Surgery, Minimally Invasive Surgery Center, San Marco Hospital GSD, Bergamo, Zingonia Italy; 7 General Surgery 3 O.U, Molinette Hospital, University Hospital Città Della Salute E Della Scienza Di Torino, Turin, Italy

**Keywords:** Indocyanine green, Fluorescence, Biopsy, Laparoscopy, Lymphoma

## Abstract

**Abstract:**

To date, no reports have indicated laparoscopic lymph node biopsies using Indocyanine green (ICG) in cases of lymphoproliferative disease. Preliminary data of patients undergoing fluorescence-guided laparoscopic lymph node biopsy (FGLLB) using ICG was retrospectively analysed from the multicentre registry FLABILY study. Between June 2022 and February 2024, 50 patients underwent FGLLB. The surgical biopsy aimed to re-stage lymphoproliferative disease for 25 patients and to establish a diagnosis in 25 patients. The median duration of the procedure was 65 ± 26.5 min. All the procedures were performed laparoscopically. One surgical conversion occurred due to bleeding. Median length of hospitalization was 1 ± 1.7 days. Two unrelated complications occurred in the immediate postoperative course. ICG was administrated preoperatively by means of an inguinal, perilesional, or intravenous injection according to the anatomical sites of the biopsy. Fluorescence was obtained in 43/50 (86%) of patients. A significant difference was highlighted in the appearance of fluorescence in sub-mesocolic lymph nodes compared to supra-mesocolic and mesenteric lymph nodes (41/49 (83.6%) vs. 13/22 (59%), *p* = 0,012). In 98% of cases, FGLLB provided the information necessary for the correct diagnosis. Fluorescence with ICG offers a simple and safe method for detecting pathological lymph nodes. FGLLB in suspected intra-abdominal lymphoma can largely benefit from this new opportunity which, to date, has not yet been tested. Further studies with a larger case series are needed to confirm its efficacy.

**Graphical abstract:**

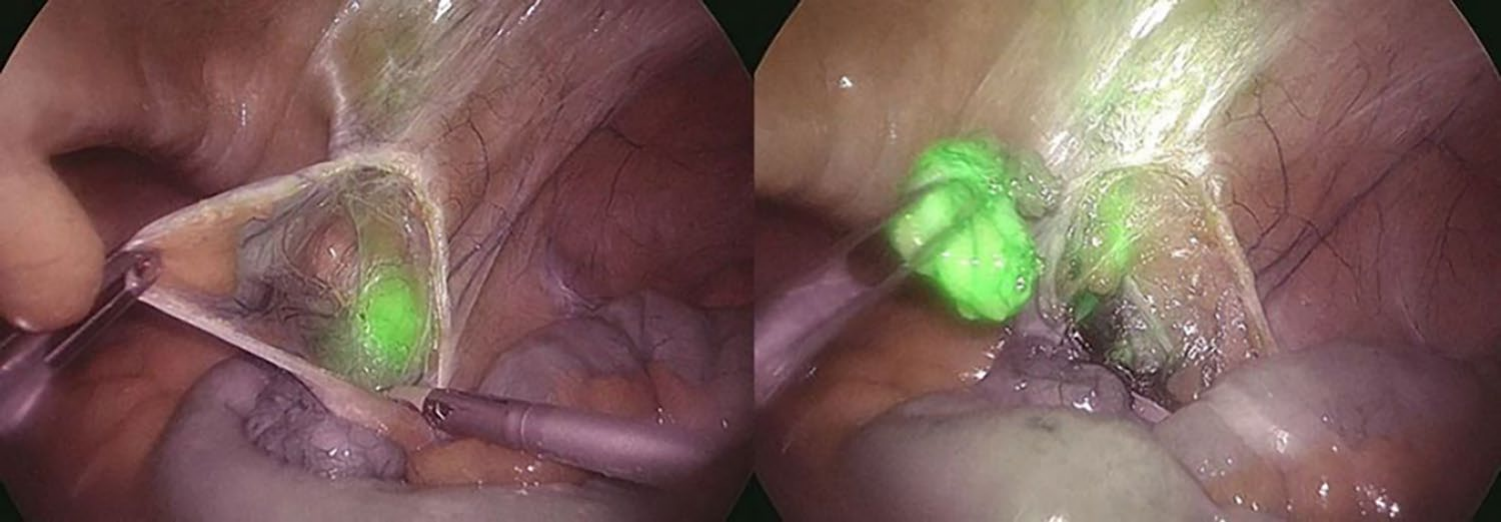

## Introduction

Among all chromophores and fluorophores that could work as probes in medical imaging techniques, near-infrared (NIR) fluorescence imaging with Indocyanine green (ICG) is emerging as a major contributor to intraoperative surgical decisions with various applications having already been described in the literature.

Recently, the use of fluorescence imaging, ICG in particular, has expanded exponentially. This has included surgery to detect tumours and sentinel lymph nodes involving the breast, lungs, liver, colon, stomach, and pelvis, as well as to assess tissue perfusion involving the viscera [1–7]. Notwithstanding the countless fields of application, it was decided to study the use of this fluorophore in elective tropism for lymph nodes (LNs) and in lymphatic tissue.

Laparoscopic lymph node biopsy (LLB) in cases of abdominal lymphoma diagnosis represents a valuable tool with excellent diagnostic accuracy. This approach safely provides adequate tissue for full histologic evaluation and is superior to needle biopsy [8].

In fact, LLB is essential to establish a diagnosis with adequate classification of the lymphoma and to follow the progression of the disease. The limitations of this technique may arise from finding small lymph nodes in difficult or anatomically challenging sites such as in the vicinity of large vessels or hollow viscera.

To date, only a few reports on the use of ICG in the surgical biopsy of pathological lymphatic tissue in cases of suspected lymphoproliferative disease [9, 10] have been done. For this reason, the multicentre FLABILY (Fluorescence-guided Laparoscopic Biopsy in Lymphoma) study was launched in June 2022 under the aegis of the SICE (Italian Endoscopic Surgery Society).

This ongoing study aims to investigate the technique of fluorescence-guided laparoscopic lymph node biopsy (FGLLB) in cases of abdominal lymph nodes affected by primary lymphoproliferative disease. The hypothetical advantage, since not yet fully studied, is to simplify the search for the lymph nodes themselves and to be able to more accurately choose the site of biopsy which needs to be located on vital tissue to obtain a contributory pathological examination.

## Methods

### Aim and objectives

The FLABILY study aims to investigate the use of ICG in cases of abdominal lymph nodes affected by primary lymphoproliferative diseases. In particular, the primary objective is to evaluate whether the elective tropism of the dye towards the lymph node is also maintained in cases of LN primary disease as demonstrated in metastatic LNs. The secondary objective is to evaluate the ideal injection site, dosage, and timing of ICG administration via the appearance of fluorescence in the pathologic LNs.

In June 2022, the FLABILY observational ongoing study was formally launched under the aegis of SICE (EudraCT registration number 2022–002639-76). Participation in the study is on a voluntary basis. Data from five Italian centres were prospectively collected in a registry using a specific database developed with MS Access (Microsoft Corporation, Redmond, WA, USA) and is available for download at https://siceitalia.com/studio-flabily-fluorescence-guided-laparoscopic-biopsy-in-lymphoma/. Patients were studied via demographic data, operative indications, type of approach and technique, and intra- and post-operative complications. Inclusion criteria were age ≥ 18 years old, absence of pathological LNs accessible to a superficial surgical biopsy and indication for abdominal LNs sampling to establish or confirm a diagnosis of the lymphoproliferative disease. Exclusion criteria were known allergies to iodides, coagulopathy, and pregnancy. Patients were studied with a preoperative positron emission tomography/computed tomography (PET/CT) scan with which the pathological LNs, their location and, above all, the degree of standardized uptake value (SUV) was assessed. We mainly identified three abdominal anatomical areas: sub-mesocolic, supra-mesocolic, and mesenteric.

In the first area, the LNs located below the transverse mesocolon were identified and mainly located in the periaortic region, from the duodenal jejunal flexure to the aortic bifurcation; it also included the LNs located along the common iliac, external iliac and internal iliac artery as well as the paracaval LNs. In the supra-mesocolic area, the periaortic LNs of the celiac trunk and its hepatic, gastric, and splenic arterial axes, the retroportal and pancreatic axes were included. The mesenteric area included the LNs throughout the mesenteric lining of the small intestine, from the Treitz ligament to the cecum. The indicators of the procedure outcome taken into consideration were the operative time, the estimated blood loss, the associated interventions, the surgical conversion, the insertion of additional trocars, the positioning of an abdominal drain, the length of hospital stay, and the post-operative complications according to Clavien–Dindo Classification [11].

Also considered was the use of the ICG in fluorescence-guided surgery, the drug dosages, the injection site of the drug and the number of patients with fluorescence obtained after ICG administration to evaluate the effectiveness. Informed consent for the procedure and the use of the dye were obtained from each patient in the study. The study was carried out according to the relevant guidelines and regulations (Declaration of Helsinki).

### Surgical procedure

All surgical procedures consisted of a minimally invasive biopsy of lymph node tissue under fluorescence guidance, retrieving a lymph node “in toto” or a fragment of a lymph node packet or lymphomatous plaque in cases of pathological extra lymph node lymphatic tissue. The biopsy was targeted on the site with the highest SUV. A dedicated clinical endoscopic system (Visera Elite II, Olympus Medical Systems Corp., Tokyo, Japan) equipped with an infrared (IR) light source and an IR UHD telescope was used to illuminate the regional lymph nodes. The surgical technique has been previously detailed and described in our previous article [12]. A solution obtained by diluting a 25 g vial of indocyanine powder with 10 ml of sterile water was used. The site and route of administration of the indocyanine green dye varied with the anatomical site of the sample. In sub-mesocolic LNs, the injection of ICG was made in both the inguinal regions of the patient in the intradermal position and intra-nodally, if possible. Half an hour to several hours before surgery 1.5–2 cc (3.75–5 mg) of the solution described above was injected per side to allow the dye, following the lymphatic flow, to penetrate the abdomen along the paraortic chains.

In the supra-mesocolic LN, a 2 ml injection of the aforementioned solution was intraoperatively given in the peritoneum near the area where the pathological lymph nodes were located. During surgery, in cases of a lack or unsatisfactory fluorescence in the site where the pathological LNs should be recognizable, an intravenous administration of 1–2 cc of the previously described solution was carried out and the appearance of fluorescence was assessed.

### Statistical analysis

The results are expressed as mean ± SD. All comparisons between the groups were performed using the Student *t* test or the Mann–Whitney *U* test. The *X*^2^ test was used to assess the relationships between categorical variables.

## Results

Data from 50 patients undergoing FGLLB surgery were retrospectively analysed. The characteristics of the patients are described in Table 1. The aim of the surgical biopsy was to re-stage the lymphoproliferative disease for 25 patients and to establish a diagnosis in 25 patients. The median duration of the procedure was 65 ± 26.5 min. All the procedures were performed laparoscopically and five of them were robotically assisted. A surgical conversion occurred in one patient due to bleeding. Blood loss was nil except for the converted patient. In most of the cases (70%), three trocars were used whilst a fourth trocar was added in 15 patients.

**Table 1:** Patients’ main characteristics

Sex, F/M	22/28
Age, year	65 (14.1)
BMI, kg/ m^2^	25.7 (4.5)
Aim of the biopsy: restaging /diagnosis	25/25
Duration of surgery, min	65 (26.5)
Trocar addition, patients (%)	15 (30)
Surgery conversion, patients (%)	1 (2)
Drain positioning, patients (%)	4 (8)
Type of biopsy: incisional/excisional/both	10/31/9
Hospital stay, days	1 (1.7)
Morbidity, patients (%)	2 (4)

Values are meant as median (SD) unless indicated otherwise

*BMI*, Body mass index;

Surgical intervention for all patients consisted of a laparoscopic biopsy of the lymphatic tissue and performing either an incisional biopsy or an excisional biopsy depending on the appearance of the pathological tissue.

The median length of hospitalization was 1 ± 1.7 days with the majority of the patients (62%) being discharged the day following the procedure. No day hospital procedures were carried out. Two unrelated Clavien–Dindo class III complications occurred in the immediate postoperative course. A prolonged postoperative stay of 11 days was due to the development of a complete ureteral obstruction as a result of tumoral compression requiring a nephrostomy and a stent. A patient with severe respiratory insufficiency had postoperative distress necessitating prolonged ventilatory assistance in the intensive care unit.

**Fig.1:**
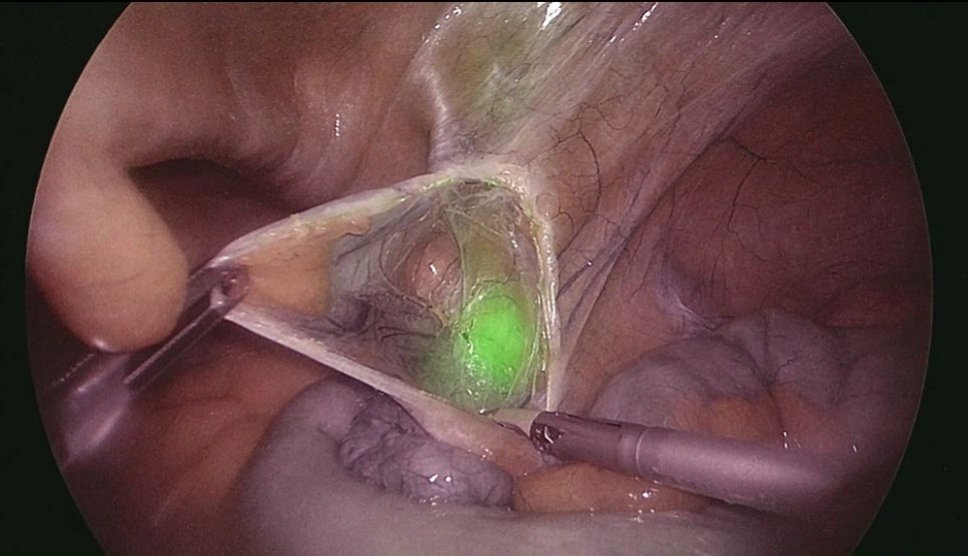
Fluorescent spot at the inner inguinal ring under near-infrared view

**Fig.2:**
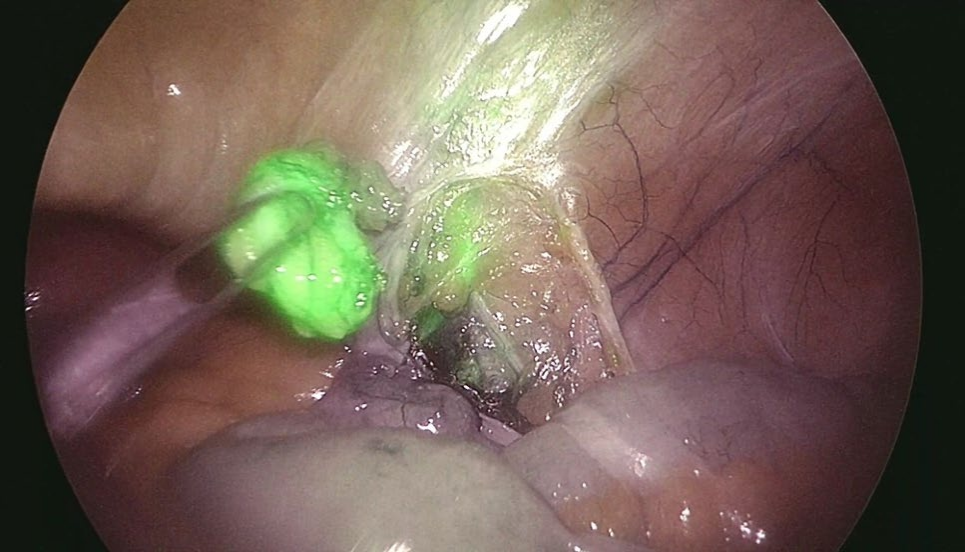
A pathologic lymph node is dissected and retrieved

Based on the anatomical location of the pathological LNs to be biopsied, patients were divided into two groups: a subLN group, including the periaortic and periliac LNs, and a supraLN group when the biopsy site was supra-mesocolic or mesenteric. Thirty-six patients were in the subLN group whereas 14 patients were in the supraLN group. The preoperative injection of ICG was mostly carried out at the inguinal level in the subLN group, whereas the inguinal and intravenous injections were numerically equivalent to those in the supraLN group. At NIR view during surgery, fluorescence of pathologic LNs was obtained in 43/50 (86%) patients (Figs. 1–2).

Table 2 highlights the difference in the appearance of the lymph node fluorescence after injection in the different areas according to the subgroup of patients. Thirteen patients in the subLN group received an injection of indocyanine on more than one site: inguinal and intravenous. Eight patients in the supraLN group received ICG on more than one site: perilesional, inguinal, or intravenous. A significant difference was highlighted in the appearance of lymph node fluorescence in subLN patients compared to supraLN patients (41/49 (83.6%) vs. 13/22 (59%), *p* = 0,012*).* Regarding the timing of ICG administration, 21 patients received an inguinal injection of ICG half an hour before surgery, 12 patients 14 to 16 h before surgery, and 8 patients 4 to 6 h before surgery. A fluorescence in pathologic LNs was obtained in 15, 10, and 8 patients, corresponding respectively in 71.4%, 83.3%, and 100% of the patients. The histopathological diagnosis was non-Hodgkin lymphoma in 35 patients, LN with nonspecific inflammatory reaction/hyperplasia in 6 patients, metastasis from solid tumours in 4 patients, Hodgkin lymphoma in 2 patients, and Sarcoidosis, Castleman disease and liponecrotic adipose tissue with inflammatory infiltrate in 1 patient, respectively. In 98% of the cases, FGLLB provided adequate specimens of lymphatic tissue and a correct diagnosis with subsequent therapeutic decisions being achieved.

**Table 2:** Appearance of lymph node fluorescence after injection in different sites according to the subgroup of patients

	SubLN patients (*n* = 36)	SupraLN patients (*n* = 14)	*p*-Value
Sites of injection			
Inguinal injection, *n* (%)	29/34 (85.3)	5/8 (62.5)	0.08
Perilesional injection, *n* (%)	NCO	1/4 (25)	
Intravenous injection, *n* (%)	12/15 (80)	7/10 (70)	0,292
All the sites, *n* (%)	41/49 (83.6)	13/22 (59)	***0,012***

Statistically significant p-values are displayed in bold

*SubLN* sub-mesocolic lymph nodes; *SupraLN* supra-mesocolic and mesenteric lymph nodes; *NCO* not carried out

## Discussion

The FLABILY study aims to investigate the use of ICG in cases of abdominal lymph nodes affected by primary lymphoproliferative diseases. The hypothetical advantage is to simplify the search for the lymph nodes themselves and to be able to more accurately choose the site of biopsy. In cases of suspected lymphoproliferative disease when superficial lymph nodes are not accessible, a laparoscopic lymph node biopsy must be performed to obtain a diagnosis. This technique is widely validated and represents a procedure well tolerated in patients with almost zero morbidity. [13, 14].

Analysis of our case series of FGLLB demonstrates that this technique seems to be equally tolerated due to: the short duration of the procedure, a conversion rate of 2.2%, discharge the day after the procedure in the majority of cases, and negligible complications. Consequently, we have verified that ICG retains its elective tropism for lymphatic tissue such that regardless of the site of administration fluorescence of the pathologic LNs appears in 86% of the cases.

Although the same dilution of the ICG solution and the same quantities injected were used in all of the cases, two parameters of the study were variable by necessity: the injection site of the ICG solution and the timing of its administration. Regarding the dye site of injection, this was based on the site of the pathological lymphadenopathies that had to be biopsied. In cases of sub-mesocolic pathologic LNs, the bilateral inguinal administration site was chosen according to a consolidated technique used for the sentinel lymph node and pelvic lymphadenectomy for melanoma as well as for the treatment of lymphedema of the lower limbs [15–17].

For mesenteric or supra-mesocolic LNs, the injection was performed perilesionally in the subserosal layer, borrowing from the lymph node mapping technique in gastric cancer [18].

The inguinal injection proved to be very effective in staining the periliac and aortic lymph nodes up to the Treitz ligament but was insufficient in the supramesocolic ones, likely due to the lymphatic pathways of the latter being more complex. The large periaortic abdominal masses of lymphoma tissue that did not stain after inguinal injection require a separate discussion. The hypothesis in this case is twofold: it may be due to the extra-nodal masses, as they can occur in 25–40% of cases [19], which are excluded from the lymphatic circulation because they originate outside of it.

Another possibility is that the LNs that are extremely enlarged by the disease undergo anatomical destructuring that can slow or even block the flow of the dye. This situation has already been described in metastatic lymph nodes. In fact, Lucas et al. [20] compared preoperative and intraoperative ICG injection staining of LNs in colon cancer and in line with results of previous studies [21, 22], it resulted in a significantly higher rate of detected LNs in the former (30.1% vs. 14.1%, *p* =  < 0.001).

LN metastases can occlude lymphatic pathways in advanced settings [23]. Allowing the dye more time to travel through the lymphatic system to reach more distant or slower-connected LNs before detection enables more effective LN mapping.

In the literature, there is no agreement on the timing of ICG administration when performing a fluorescence lymphography through an inguinal injection and it varies from immediately before surgery to several hours before [15–17]. Our study protocol requires that it be carried out at various times pre-operatively in the hours preceding the operation.

In our study, we had the opportunity to clinically verify the effects of slowing the lymphatic flow and therefore of the appearance of fluorescence in the pathologic lymph nodes, which is why the timing of administration was also subject to variability. An ideal timing of administration was sought by varying between the immediate pre-operative injection and the one carried out 12 h before, with the conclusion being that an injection carried out 4 to 6 h before could include the greatest number of stained lymph nodes. There are still open questions regarding dosages, quantities, and administration sites of ICG in many surgical procedures. The degree of lymph node disease represents an additional condition that has been studied in a very limited capacity but is capable of influencing the fluorescence of the lymph node. The lack of randomized trials on these topics means that to date there is no consensus.

As a matter of fact, following the rapid development of surgical techniques that involve the use of ICG, most dosage and timing information is based on recommendations collated from worldwide surgical experts in these procedures and is not evidence-based.

## Conclusions

ICG enhanced fluorescence seems to provide several advantages in FGLLB allowing for both better clarification of the surgical anatomy and quicker identification of the structures to be biopsied. These advantages are more evident especially if the pathologic LNs are located in the sub-mesocolic area. This allows for a more precise dissection because it is aimed at a visible target and therefore allows for a safe dissection.

The FLABILY study should respond to unsolved issues and give weight to the encouraging preliminary results, which is a necessary step prior to considering this novel application of ICG in FGLLB as completely reliable.

## Data Availability

The datasets generated during and/or analyzed during the current study are available from the corresponding author upon reasonable request.
